# Estado actual y retos futuros de la medicina de laboratorio en España: un análisis de la Sociedad Española de Medicina de Laboratorio (SEQC^ML^)

**DOI:** 10.1515/almed-2022-0108

**Published:** 2022-12-13

**Authors:** Imma Caballé, Antonio Buño, Francisco A. Bernabeu, Francesca Canalias, Antonio Moreno, Mercè Ibarz, José Puzo, Concepción González, Álvaro González

**Affiliations:** Laboratorio Catlab, Terrassa, España; Servicio de Análisis Clínicos, Hospital Universitario La Paz, Madrid, España; Servicio de Bioquímica–Análisis Clínicos, Hospital Universitario Puerta de Hierro Majadahonda, Madrid, España; Laboratori de Referència d’Enzimologia Clínica, Departament de Bioquímica i Biologia Molecular, Universitat Autònoma de Barcelona, Cerdanyola del Vallès, España; Servicio de Análisis Clínicos, Hospital de Meixoeiro, Vigo, España; Laboratorio Clínico ICS Lleida, Hospital Universitario Arnau de Vilanova de Lleida, Lleida, Spain; Servicio de Análisis Clínico y Bioquímica, Hospital Universitario San Jorge, Huesca, España; Servicio de Bioquímica Clínica, Hospital Universitario Virgen Macarena, Seville, España; Laboratorio de Bioquímica, Clínica Universidad de Navarra, Pamplona, Spain

**Keywords:** medicina de laboratorio, estado sectorial, retos futuros

## Abstract

**Objetivos:**

La medicina de laboratorio es una disciplina clave que contribuye al diagnóstico, terapia y seguimiento adecuado de los pacientes. Actualmente se enfrenta a grandes retos debido a la innovación tecnológica y al aumento de la demanda. Desafortunadamente hay una información limitada para comprender la situación actual en España. Este estudio pretende mostrar la situación de los laboratorios clínicos y de los profesionales de la medicina laboratorio.

**Métodos:**

Desde la Sociedad Española de Medicina de Laboratorio se envió una encuesta a los 250 centros más representativos (mayor volumen y con programas formativos), de los cuales 174 (69,6%) respondieron con datos referidos a 2019.

**Resultados:**

Los laboratorios se clasificaron en función del número de determinaciones. El 37% se consideraron pequeños (<1 millón de determinaciones al año), el 40% medianos (entre 1 y 5 millones de determinaciones al año) y el 23% grandes (>5 millones de determinaciones al año). El nivel de especialización de los facultativos y la eficiencia fueron mayores en los laboratorios grandes. La mayoría de las peticiones (87%) y determinaciones (93%) se concentraron en Bioquímica y Hematología. En relación con los facultativos, el 63% disponían de contrato fijo y el 23% superaban los 60 años.

**Conclusiones:**

La medicina de laboratorio en España es una disciplina consolidada y en crecimiento, capaz de aportar valor al diagnóstico, pronóstico y seguimiento de enfermedades y sus tratamientos. El conocimiento del estado actual nos permitirá abordar retos tales como la necesidad de formación de profesionales, la innovación tecnológica, la aplicación del Big data, la optimización de los sistemas de gestión de calidad y la seguridad del paciente.

## Introducción

La Medicina de Laboratorio se puede definir como una disciplina clínica, dedicada a la medición cuantitativa, o evaluación cualitativa, de cualquier sustancia que pueda ser ensayada en cualquier tipo de fluido biológico, con fines médicos o de investigación. Dicha medición o evaluación se suele referir como diagnóstico *in vitro* dado que se realiza fuera del organismo [1]. Los resultados de estas mediciones se traducen en información que sirve para mejorar la salud y el bienestar de los individuos y de la población [2, 3]. Los resultados de los laboratorios clínicos sirven para cribado de las enfermedades, a su prevención y detección temprana, al diagnóstico, al seguimiento y la predicción de la respuesta al tratamiento. Se estima que en torno al 66% de las decisiones clínicas se basan en los resultados de los laboratorios clínicos [4, 5].

Pese al gran valor e importancia de los análisis de laboratorio en el diagnóstico y manejo de los pacientes, el gasto sanitario que representa es limitado, alrededor del 3% en España [6]. Los laboratorios clínicos se enfrentan a una creciente demanda de determinaciones debidas al incremento en la incidencia en las enfermedades crónicas e infecciosas y a el envejecimiento de la población [7]. En Europa el mercado de los laboratorios clínicos alcanzó los 13.825 millones en 2019 y se estima un crecimiento de más del 4,5% en los próximos años hasta superar los 18.000 millones en 2027 [7].

La información sobre la situación actual de los laboratorios clínicos en España es escasa, como demuestra la falta de una base de datos oficial. Este estudio pretende abordar el estado actual de los laboratorios clínicos en España como primer hito para definir estrategias y acciones que mejoren su funcionamiento en el futuro.

El objetivo de este estudio es recopilar información sobre la actividad de los laboratorios clínicos en España, describir la cartera de servicios y las áreas de mayor demanda, y describir la situación de los profesionales del sector. Se pretende así mismo divulgar sobre la investigación y docencia impulsadas desde los laboratorios clínicos Desde la Sociedad Española de Medicina de Laboratorio se impulsó esta iniciativa que se concretó en la elaboración del Libro Blanco de la Medicina de Laboratorio. Este documento representa una primera aproximación al estado de situación y en este artículo se muestran los resultados más destacados [8].

## Materiales y métodos

### Selección de centros

Para construir una imagen general de los laboratorios clínicos se seleccionaron los 250 laboratorios más representativos del listado aportado por el Ministerio de Sanidad, Consumo y Bienestar Social en enero de 2020 [9]. Se consideraron más representativos aquellos centros incluidos en el Catálogo Nacional de Hospitales, priorizando los grandes centros hospitalarios (aquellos con mayor número de camas) (Figura Suplementaria) y que cuentan con capacidad docente. Se hizo una encuesta a todos los centros seleccionados.

### Variables de interés

Se recogieron datos del año 2019, durante el cual se han informado 55,9 millones de peticiones y 800 millones de determinaciones. Se descartó el uso de datos de años posteriores para evitar distorsiones debidas a la pandemia de la COVID-19.

Se recogieron datos generales del laboratorio (servicios, titularidad), información del modelo organizativo y de actividad (número de peticiones, modelo de atención continuada, adquisición de equipos o fungibles), datos sobre recursos humanos (tipologías de contratos, demografía de los empleados), información sobre el tipo de docencia (número de docentes y residentes en formación), sobre los sistema de calidad adoptados, la naturaleza de las investigaciones lideradas por el laboratorio (tesis doctorales, trabajos fin de máster o grados) y el grado de automatización de los procesos. Se cuestionó a los participantes sobre los retos y prioridades futuras detectados a su criterio.

Para establecer un punto de comparación externo al presente análisis se utilizaron datos del informe de la Federación Española de Empresas de Tecnología Sanitaria (FENIN) 2018 [10] datos del ministerio [11, 12] y datos globales [13].

Este estudio cumplió con la Ley Orgánica 3/2018 de Protección de Datos Personales y garantía de los derechos digitales. Todos los datos se trataron de forma confidencial y anónima.

### Análisis estadístico

Las variables cualitativas se muestran como números totales y porcentajes. Las variables cuantitativas se muestran representadas como media o mediana e intervalos intercuartílicos. El análisis estadístico se llevó a cabo mediante Excel con macros y *add-ins* customizados.

## Resultados y discusión

### Estructura y modelo de los laboratorios clínicos en España

**Figura 1:**
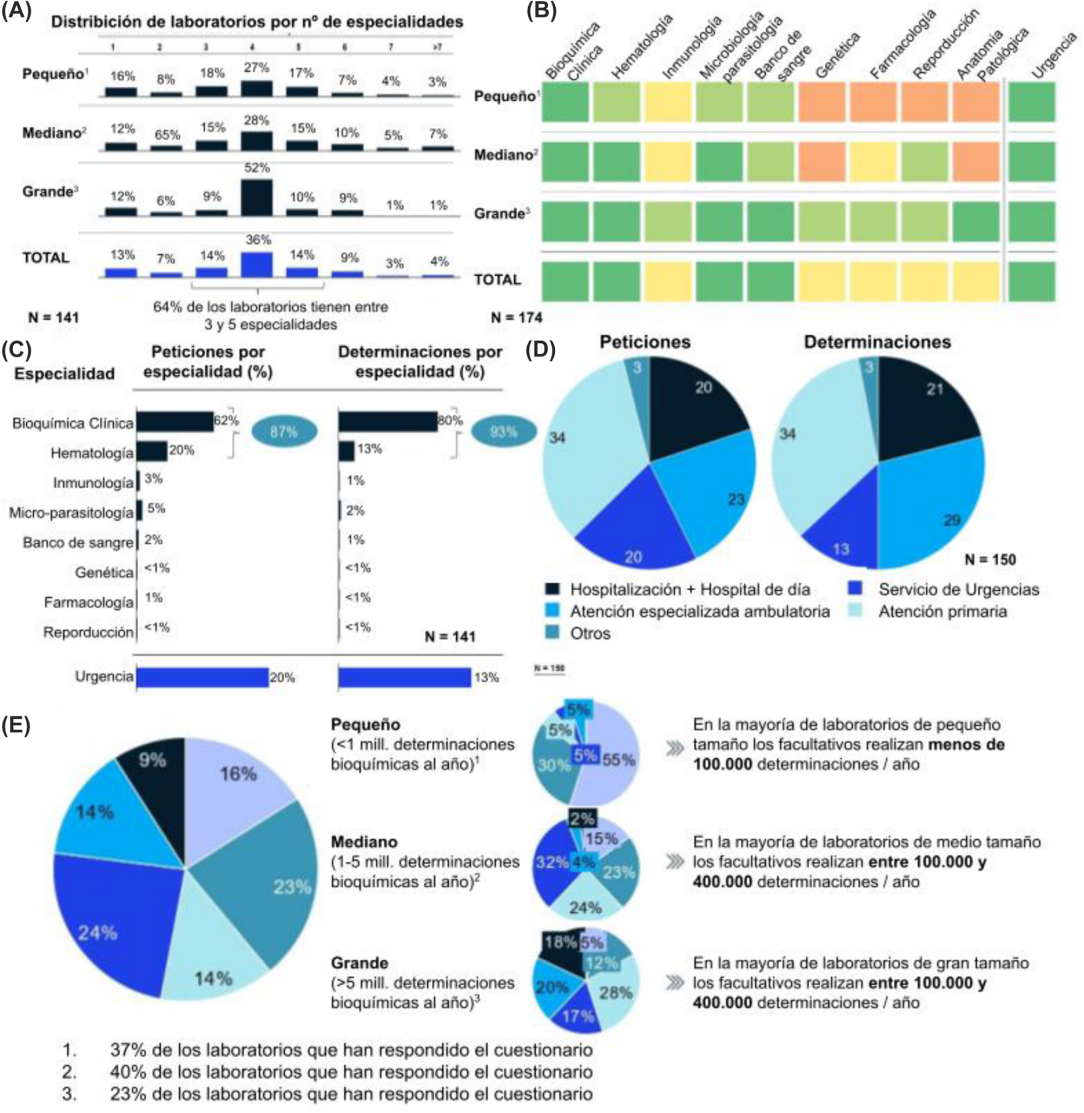
Modelo del sector y actividad desarrollada. (A) Porcentaje de laboratorios con existencia de un número determinado de especialidades, (B) especialidades existentes en los Laboratorios de Análisis Clínicos según su tamaño y existencia de servicio de urgencias, (C) porcentaje de peticiones y determinaciones por especialidad, (D) peticiones y determinaciones por procedencia, (E) distribución de los Laboratorios Clínicos según el número de determinaciones por facultativo.

De los laboratorios encuestados, el 66% eran de tipo general, y el 90% disponían solo de un centro hospitalario asociado. El 37% de los laboratorios se consideran pequeños en relación con el volumen de determinaciones (<1 millón de determinaciones al año), el 40% medianos (entre 1 y 5 millones de determinaciones al año) y el 23% grandes (>5 millones de determinaciones al año). La mayoría de los laboratorios disponían entre 3 y 5 especialidades, siendo mayor el número de especialidades en los centros pequeños (Figura 1A). Esto podría indicar distintos modelos organizativos en función del tamaño del laboratorio y que los laboratorios de mayor volumen están más orientados a la realización de test diagnósticos a gran escala. Bioquímica, Hematología, Microbiología y Banco de sangre están disponibles en la mayoría de los centros (>60%), y en torno a la mitad de los centros disponen de inmunología, farmacología, genética, andrología y reproducción y anatomía patológica (Figura 1B). Un 80% disponen de servicio de urgencias integrado en el laboratorio.

### Actividad desarrollada por los laboratorios clínicos en España

En cuanto a la actividad por especialidad, bioquímica clínica y hematología concentraron la mayor parte de peticiones (87%) y determinaciones (93%). El 20% de las peticiones y el 13% de las determinaciones fueron urgentes (Figura 1C). La mayor parte de las peticiones y determinaciones vinieron de atención primaria (34% para ambas) y atención especializada (23% y 29% para peticiones y determinaciones respectivamente), siendo éstas últimas las peticiones con mayor número de determinaciones con una media de 16 determinaciones por petición (Figura 1D). Las peticiones de atención primaria contaron con una media de 15,5 determinaciones. La eficiencia del personal, medida en función del número de determinaciones por facultativo, fue mayor en los laboratorios grandes. Un 95% de los laboratorios grandes realizan entre 100.000 y 400.000 determinaciones por año y facultativo (Figura 1E). Es posible que estos centros que realizan más test a gran escala se beneficien de una mayor especialización de sus facultativos que lleve a una mayor eficiencia. En global, los laboratorios clínicos mostraron una capacidad de cubrir internamente el 97% de las determinaciones pedidas. Esto demuestra una ligera necesidad de aumentar la capacidad de los laboratorios clínicos para acabar de satisfacer toda la demanda.

### Prácticas de gestión, estrategia, compra y contratación

**Figura 2:**
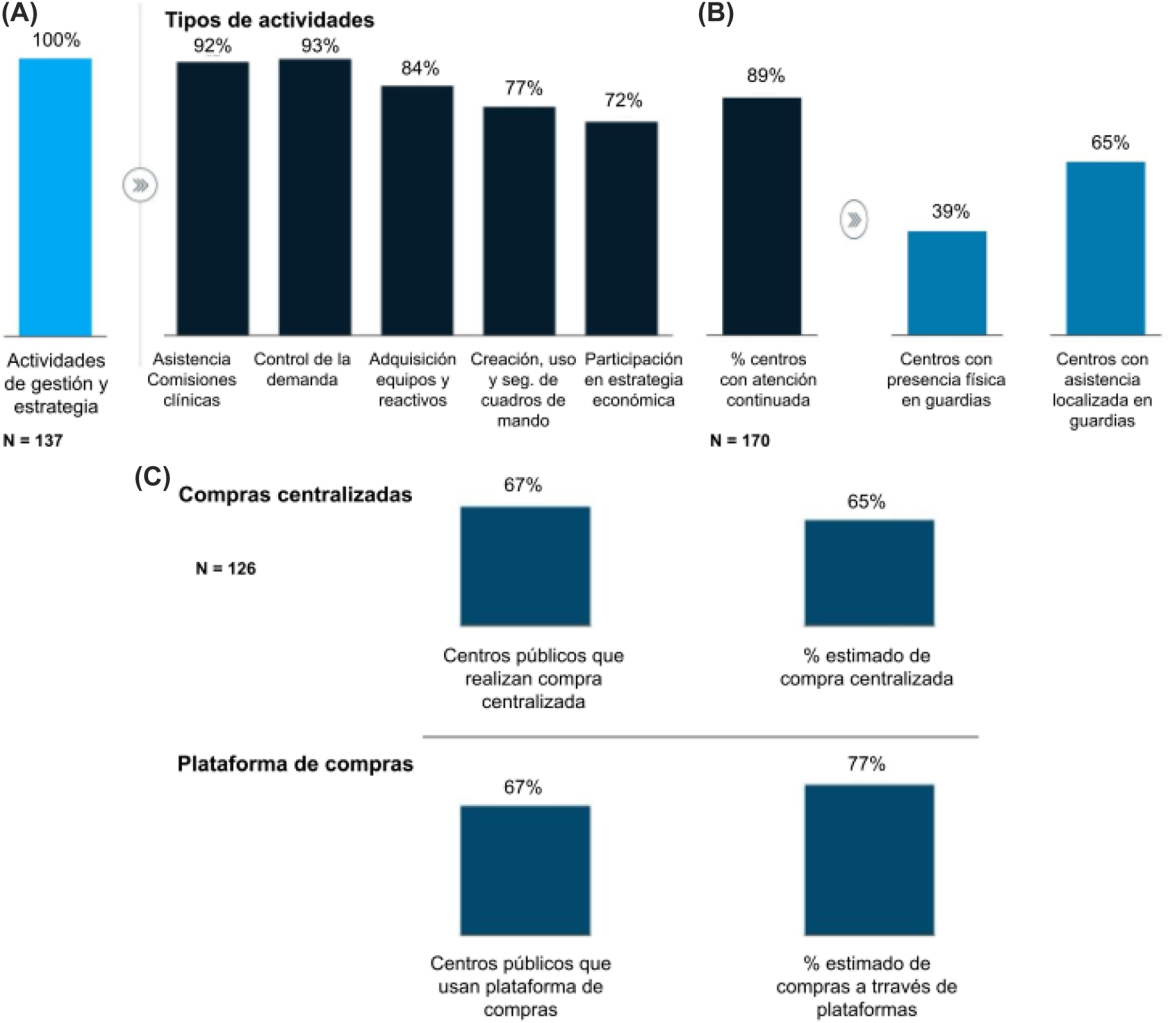
Prácticas de gestión, estrategia, compra y contratación. (A) Porcentaje de laboratorios que realizan actividades de gestión y estrategia y desglose por principales actividades de gestión y estrategia, (B) modelo de atención continuada, (C) nivel de centralización de las compras y uso de plataformas de compras.

Todos los laboratorios realizaron actividades de gestión o estrategia, siendo las más comunes la asistencia a comisiones clínicas (92%) el control de la demanda (93%), y la adquisición de equipos y reactivos (84%) (Figura 2A). El 89% de los laboratorios disponían de un modelo de atención continuada, con asistencia localizada en las guardias (65%) o presencia física (39%) (Figura 2B).

Un total de 126 centros, todos ellos públicos, realizaron de forma rutinaria compras centralizadas a través de plataformas, estimando que un 65% sobre el total de compras se gestionó de forma central (Figura 2C).

### Recursos humanos

**Figura 3:**
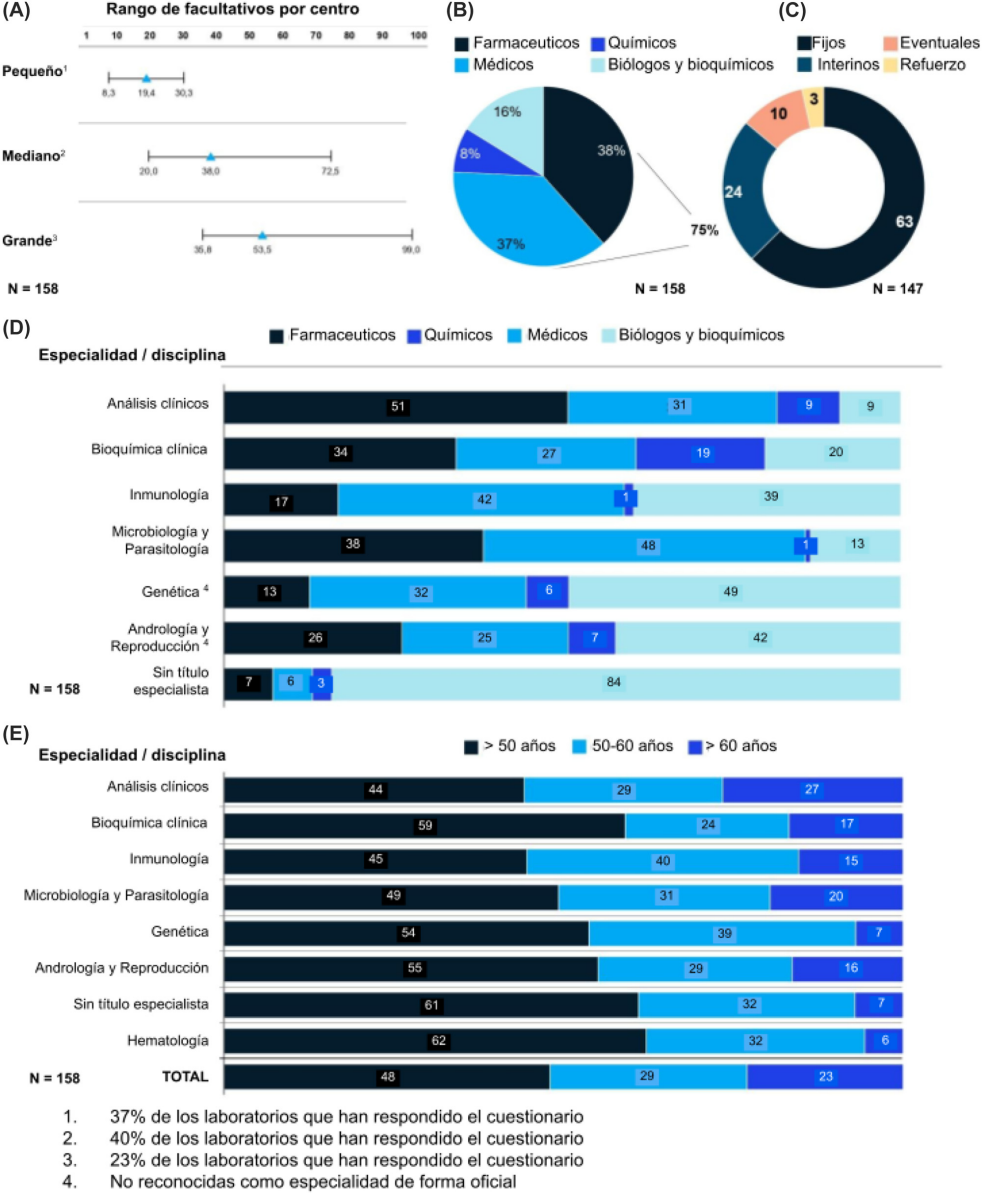
Recursos humanos. (A) Número de facultativos por laboratorio por tamaño, según datos del cuestionario cumplimentado por los participantes, (B) distribución de tipos de contrato en los centros participantes en el cuestionario, (C) distribución de titulados, (D) distribución de titulados por área de conocimiento, (E) distribución de segmentos de edad de titulados superiores por especialidad.

El número de facultativos por centro aumenta proporcionalmente al tamaño del centro (Figura 3A). El 63% de los facultativos disponían de un contrato fijo. Un 24% eran interinos, un 10% eventuales y un 3% de refuerzo (Figura 3B). La contratación presentó diferencias territoriales. Cataluña, Navarra, Asturias y Galicia son las Comunidades Autónomas con mayor porcentaje de facultativos con contrato fijo, superando el 70%. En general, un porcentaje relativamente alto de profesionales desempeñan su actividad a tiempo completo.

En cuanto a su formación, el 38% de los facultativos son graduados en Farmacia, el 37% en Medicina, el 16% en Biología y Bioquímica y el 8% en Química (Figura 3C). Médicos y farmacéuticos prevalecen en las especialidades clásicas más abundantes como análisis clínico (82%) o bioquímica (61%), mientras biólogos y bioquímicos lo hacen en áreas como genética (49%), andrología y reproducción (42%) u otras (84%) (Figura 3D).

En relación con la edad de los facultativos en España, se observó un 23% de titulados que superan los 60 años, lo que implica antes de 2024 la necesidad de renovar en torno al 20–25% de las plazas (Figura 3E).

### Formación continuada, docencia e investigación

**Figura 4:**
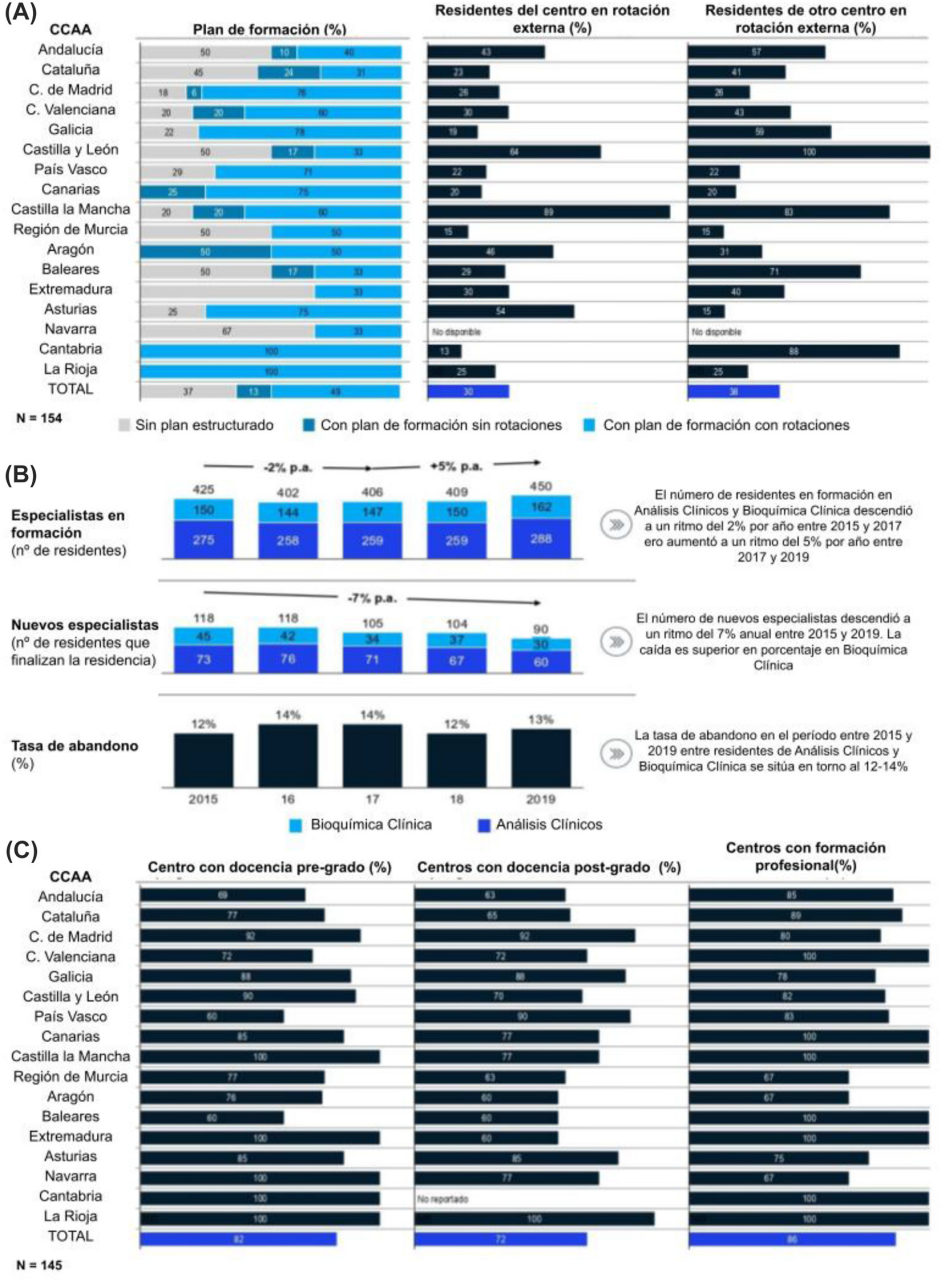
Formación continuada, docencia e investigación. (A) Porcentaje por Comunidad Autónoma de centros con planes de formación estructurados, Porcentaje de residentes rotando por Comunidad Autónoma y porcentaje de residentes de fuera del centro haciendo rotaciones en el mismo por Comunidad Autónoma, (B) especialistas en formación, nuevos especialistas y tasas de abandono en Análisis Clínicos y Bioquímica Clínica, (C) porcentaje de Laboratorios Clínicos que imparten docencia relativa a pre-grado, post-grado y FP por Comunidad Autónoma.

La mayoría de los Laboratorios Clínicos (63%) tienen un plan de formación estructurado con rotaciones (50%) o sin ellas (13%) (Figura 4A).

El número de médicos internos residentes (MIR) disminuyó un 2% entre 2015 y 2017, aunque repuntó un 5% entre 2017 y 2019 tomando como referencia los datos del sistema nacional de salud (SNS) [12]. En cuanto a los nuevos especialistas, el número ha ido disminuyendo a un ritmo del 7% anual entre 2015 y 2019, Las especialidades de laboratorio clínico fueron de las menos demandadas, y la tasa de abandono se situó entre el 12–14% (Figura 4B). Estos datos destacan el escaso poder de atracción de estas especialidades en los nuevos profesionales, lo que puede derivarse del pobre conocimiento que se tiene de este sector durante la formación universitaria y la escasa interacción con el paciente de los profesionales médicos desde el laboratorio clínico. Esta tendencia no se observó entre otras licenciaturas como biología o química.

A nivel nacional, el 82% de los centros encuestados imparten docencia pre-grado, un 72% postgrado y un 86% Formación profesional (FP). Destacan la Comunidad Valenciana, Castilla y León, Canarias, Castilla-La Mancha, Baleares, Extremadura y Cantabria, comunidades en las que el 100% de los centros encuestados imparten docencia de FP (Figura 4C). Además, el 13,5% de los facultativos de los laboratorios clínicos trabajaban como profesores en la universidad con distintas modalidades contractuales. Los laboratorios cuentan con una media de 13,5 publicaciones indexadas por docente en activo en los últimos 5 años, con un mayor número de proyectos de investigación por facultativo en los centros grandes. Esta actividad investigadora también se ve reflejada por el hecho que el 45% de los centros encuestados cuenta con algún tipo de asociación con un centro o instituto de investigación. El 18% de los centros encuestados cuentan con contratos de investigación con empresas, alcanzando una media de 3 contratos en esos centros.

La alta penetración de la docencia e investigación en los centros, tanto de pre-grado, postgrado y FP, la presencia sostenida de profesionales residentes, y la puesta en marcha de planes de formación específicos para los mismos, demuestran la importancia del conocimiento y la realización de docencia en los centros objeto del análisis. Cabe destacar, a su vez, la importancia de las rotaciones de residentes como valor añadido a los planes de formación estructurada en la mitad de los centros. La importante actividad científica reportada por los centros es un claro indicador de la impulsión de la actividad investigadora. Un alto componente de innovación resulta esencial a la hora de contar con servicios sanitarios de vanguardia que sean, a la vez, eficientes, para poder dar respuesta a problemas existentes y retos futuros.

### Marco regulatorio y calidad

**Figura 5:**
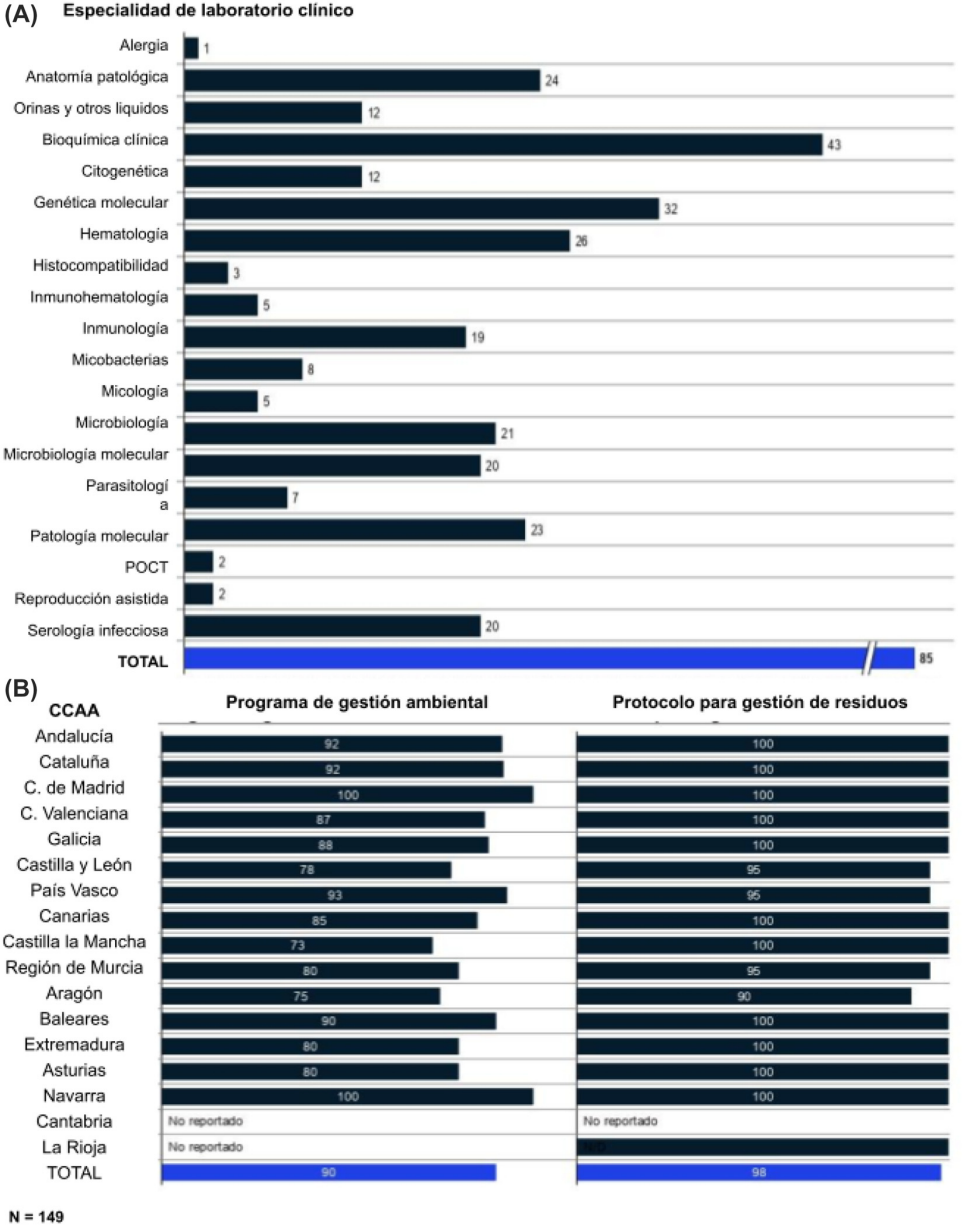
Marco regulatorio y de calidad. (A) Número de centros con acreditación UNE-EN-ISO 15189 por especialidad según la ENAC (mayo, 2021), (B) porcentaje de centros con programa de gestión medioambiental y protocolo de gestión de residuos.

Sólo el 49% de los centros que contestaron cuentan con la certificación ISO 9001. En cuanto a la acreditación en materia de sistema de Gestión de la Calidad en Laboratorios Clínicos (UNE-EN ISO 15189) en total en España solo existen 85 laboratorios según los datos de la propia Entidad Nacional de Acreditación (ENAC) (Figura 5A) [14]. Estos datos demuestran que todavía queda mucho trabajo para conseguir estandarizar los procedimientos y acreditar la calidad de los servicios en la mayoría de centros.

En cuanto a los planes de actuación relativos a medioambiente, un 90% de los laboratorios poseían un programa de gestión medioambiental y un 98% contaban con un protocolo de gestión de residuos (Figura 5B). Estos datos prueban un claro compromiso medioambiental por parte de los centros.

### La medicina de laboratorio española en el contexto europeo

El gasto medio en suministros para el diagnóstico *in vitro* en el año 2019 en los países del entorno es de 2.161 millones de Euros en Alemania, 1.623 en Francia y 859 en Reino Unido, mientras que en España fue de 1.033 millones de Euros [15]. En España el gasto ha aumentado notablemente entre 2019 y 2020. Esto sitúa España en rango con los países del entorno, con un gasto medio de 22 euros/habitante. Por el contrario, se estima que el número de especialistas por millón de habitantes es mucho mayor en España (53,3 especialistas /millón hab.) que en países del entorno como Alemania (25,5 especialistas /millón hab.). Cabe tener en cuenta diferencias en el tipo de titulaciones con acceso a las plazas de especialista, en Reino Unido y Alemania no hay plazas para farmacéuticos. Además, las diferencias en la formación de los técnicos de laboratorio también pueden tener un impacto cuando se compara el número necesario de especialistas.

### Tendencias y retos futuros

España es uno de los países donde el envejecimiento de la población es mayor, y ello tendrá impacto en la carga de morbilidad general de la población, en las enfermedades crónicas, y por ende en el incremento de la demanda de análisis clínicos. Por otro lado, los avances en biología molecular y la genómica junto con otras disciplinas científicas como la computación, análisis de datos y la inteligencia artificial están originando una Bio-revolución que sin lugar a dudas generará cambios estructurales en el modo de entender los análisis clínicos.

**Figura 6:**
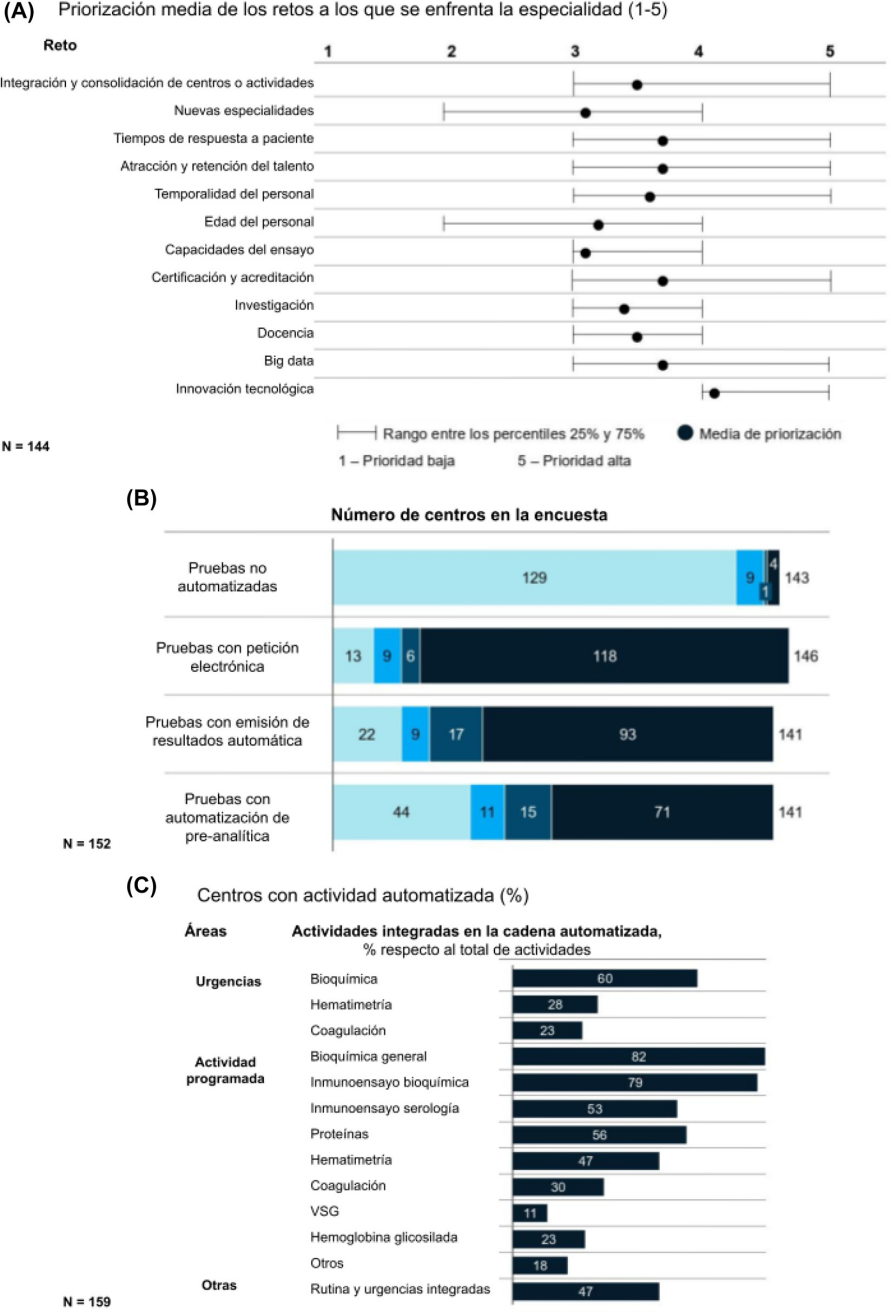
Retos futuros. (A) Principales retos a los que se enfrenta la especialidad, según cuestionario, (B) automatización de procesos por tipo de prueba, según respuestas al cuestionario, (C) porcentaje de centros con cadena automatizada según respuestas al cuestionario.

Los profesionales encuestados priorizaron como retos futuros a los que se enfrenta la especialidad: la innovación tecnológica, el análisis del Big data, la agilidad en la respuesta al paciente, los sistemas de acreditación de calidad y la atracción y retención del talento (Figura 6A). La innovación tecnológica, no solo se valora desde una óptica de eficiencia en las operaciones, sino como elemento de creación de valor para atender nuevas necesidades de los usuarios, gestionar e interpretar la gran cantidad de datos ofreciendo un servicio eficiente, seguro y cada vez más personalizado a los mismos. En este aspecto, la tecnología puede jugar un papel determinante como elemento transversal. La aplicación de *Big data* y de la Inteligencia Artificial en el Laboratorio Clínico tendrá un papel fundamental en las decisiones clínicas y monitorización de la salud, con algunas iniciativas ya en marcha [16]. De hecho, los centros encuestados han realizado una intensa labor de incorporación de tecnologías digitales y de automatización en sus modelos operativos. El 80% de los centros reciben sus datos mayoritariamente de forma electrónica, el 90% tienen la mayoría de sus pruebas automatizadas, y dos tercios (68%) cuentan con sistemas de emisión de resultados automatizados (Figura 6B and C).

## Conclusiones

Este estudio describe con mayor detalle el estado actual de la medicina de laboratorio en España y sienta las bases para potenciales acciones que permitan mejorar el sector y enfrentar los retos futuros. No obstante, el estudio representa un corte transversal, una imagen fija en el tiempo. De cara a poder inferir tendencias y poder anticipar con mayor precisión las necesidades del sector se deberían realizar estudios longitudinales.El análisis indica que la evolución de las especialidades de Análisis Clínicos y Bioquímica Clínica y la formación ha confluido y obliga a reconsiderar la situación para hacerla homologable a otros países europeos.

Los profesionales que trabajan en el Laboratorio Clínico incluyen farmacéuticos, médicos, químicos y biólogos, y no solo médicos como consta en la actualidad en las previsiones oficiales. Sectores emergentes como la genética cuentan con un elevado porcentaje de biólogos que, junto con la necesidad de afrontar la renovación de las plantillas, requerirá un aumento del número de plazas ofertadas. La necesidad de impulsar la formación especializada es una exigencia ineludible frente a la escasez de plazas.

El impacto de la COVID-19 no ha sido analizado en este estudio, pero deberá tenerse en cuenta al menos para lo relativo a los próximos años, tanto por el impacto en el número de determinaciones, como el tipo de modelo, con una tendencia actual creciente de las pruebas diagnósticas DTC (*direct to consumer*). Las pruebas POCT (*point of care testing*) pueden aumentar significativamente en un futuro cercano fruto de la evolución de las aplicaciones móviles para salud, m-Health. Existen múltiples innovaciones que tratan de dar respuesta en este sentido.

Este estudio también destaca la necesidad de disponer de un catálogo actualizado de laboratorios e incorporar los mismos a la estadística sanitaria oficial. Se requieren también avances en materia regulatoria para alinearnos con el entorno europeo y avanzar en la acreditación de calidad de los laboratorios.

La colaboración entre sociedades estatales e internacionales puede facilitar el acceso a la formación, fomentar la incorporación de jóvenes profesionales en comisiones y órganos de decisión, aumentar su acceso a becas y convenios de colaboración con Sociedades Científicas Médicas para atraer y retener nuevos talentos que potencien la innovación en el sector.

La medicina de laboratorio necesita aumentar la presencia en medios de comunicación para difundir su contribución a la sociedad en general y avanzar en el acercamiento a órganos e instituciones a nivel autonómico y nacional con el fin de lograr su apoyo en el avance y desarrollo de la disciplina y situarla en una posición más relevante dentro del sistema sanitario.

## Supplementary Material

Supplementary Material

## Material Suplementario

La versión en línea de este artículo ofrecematerial suplementario (https://doi.org/10.1515/almed-2022-0108).
