# Supplementary material for: Estado actual y retos futuros de la medicina de laboratorio en España: un análisis de la Sociedad Española de Medicina de Laboratorio (SEQCML)
Source: Adv Lab Med. 2022 Dec 13;4(1):81–91. [Article in Spanish] doi: 10.1515/almed-2022-0108 (PMC10197193; doi:10.1515/almed-2022-0108)

**MATERIAL SUPLEMENTARIO**

Figura Complementaria 1. Centros participantes por Comunidad autónoma y tipo de centro


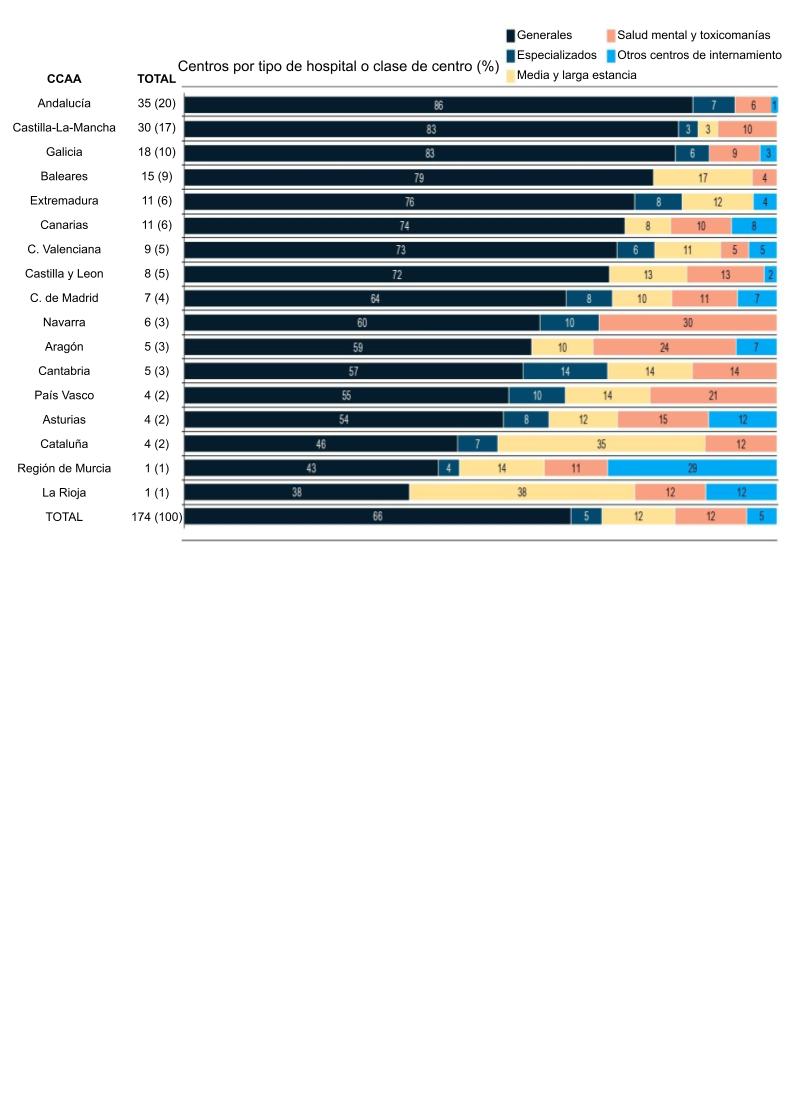

Supplement: Supplementary file 1 — Supplementary Material [file j_almed-2022-0108_suppl_001.docx]
